# Laparoscopic right-sided colon resection for colon cancer—has the control group so far been chosen correctly?

**DOI:** 10.1186/s12957-018-1417-3

**Published:** 2018-06-28

**Authors:** Jörg O. W. Pelz, Johanna Wagner, Sven Lichthardt, Johannes Baur, Caroline Kastner, Niels Matthes, Christoph-Thomas Germer, Armin Wiegering

**Affiliations:** 1 Department of General, Visceral, Vascular and Pediatric Surgery, University Hospital, University of Wuerzburg, Oberduerrbacherstr.6, 97080 Wuerzburg, Germany; 2 Comprehensive Cancer Centre Mainfranken, University Hospital, University of Wuerzburg, Josef-Schneiderstr. 6, 97080 Wuerzburg, Germany; 3 Department of Biochemistry and Molecular Biology, University of Wuerzburg, Am Hubland, 97074 Wuerzburg, Germany

**Keywords:** Colon cancer, Laparoscopic right colectomy, Lymph nodes

## Abstract

**Background:**

The treatment strategies for colorectal cancer located in the right side of the colon have changed dramatically during the last decade. Due to the introduction of complete mesocolic excision (CME) with central ligation of the vessels and systematic lymph node dissection, the long-term survival of affected patients has increased significantly. It has also been proposed that right-sided colon resection can be performed laparoscopically with the same extent of resection and equal long-term results.

**Methods:**

A retrospective evaluation of a prospectively expanded database on right-sided colorectal cancer or adenoma treated at the University Hospital of Wuerzburg between 2009 and 2016 was performed. All patients underwent CME. This data was analyzed alone and in comparison to the published data describing laparoscopic right-sided colon resection for colon cancer.

**Results:**

The database contains 279 patients, who underwent right-sided colon resection due to colorectal cancer or colorectal adenoma (255 open; 24 laparoscopic). Operation data (time, length of stay, time on ICU) was equal or superior to laparoscopy, which is comparable to the published results. Surprisingly, the surrogate parameter for correct CME (the number of removed lymph nodes) was significantly higher in the open group. In a subgroup analysis only including patients who were feasible for laparoscopic resection and had been operated with an open procedure by an experienced surgeon, operation time was significantly shorter and the number of removed lymph nodes is significantly higher in the open group.

**Conclusion:**

So far, several studies demonstrate that laparoscopic right-sided colon resection is comparable to open resection. Our data suggests that a consequent CME during an open operation leads to significantly more removed lymph nodes than in laparoscopically resected patients and in several so far published data of open control groups from Europe. Further prospective randomized trials comparing the long-term outcome are urgently needed before laparoscopy for right-sided colon resection can be recommended ubiquitously.

## Background

Colorectal carcinoma (CRC) is the most common malignancy of the gastrointestinal tract and is the third most common tumor disease with an incidence of more than 1 million per year and about 500,000 deaths per year [1]. About 40% of all CRC are located in the right hemicolon [1]. In the last decades, CRC therapy has changed drastically. After the introduction of the total mesocolic excision (TME) by Prof. Heald primarily for the rectum carcinoma, the number of local recurrences was reduced and the survival rate increased significantly [2, 3]. Similarly to the TME of the rectum, the research group of Prof. Hohenberger proposed the concept of the complete mesocolic excision (CME) for right-sided colon cancer [4]. In this case, the dissection is preformed layer-adapted with consideration of the embryonal development and central ligation of the vessels and excision of the visceral mesentery. This allows the dissection of almost all tumor-draining lymph nodes [5]. This resection technique of right-sided colon cancer has been increasingly established internationally and has led to an improved 5-year survival rate compared to the less systematic operation in a case-control study [5]. A population-based study from Denmark showed a significantly increased disease-free survival after 4 years in the group of patients operated with CME. Furthermore, significantly more lymph nodes were removed [6]. This advantage especially affects patients with low Union for International Cancer Control (UICC) stages.

Simultaneously, laparoscopic surgery is gaining in importance. Laparoscopically operated patients can be mobilized faster, regular bowel movement is re-established earlier, and the average length of hospital stay is shorter [7, 8]. Studies comparing the laparoscopic to the open resection of left-sided colon cancer show comparable oncological outcomes [9–11].

Up to date, the data regarding the laparoscopic resection of right-sided colon cancer is insufficient. Several studies have compared the laparoscopic resection with historic patient data and were able to show a non-inferiority of the laparoscopic resection [12, 13]. However, during the past years, the results for the open right hemicolectomy with CME have improved considerably showing a significantly higher number of resected lymph nodes.

In this study, we include patients who only undergo an operation due to suspected or proven right-sided colon cancer. We systematically compared the results of the laparoscopic right-sided hemicolectomy to the open operation concerning the number of resected lymph nodes as a surrogate parameter for the quality of the CME and the oncological outcome. The aim of this study was to compare if laparoscopic right-sided colon resection is equal to the amount of lymph nodes resected.

## Methods

### Data sources

This study includes all patients who underwent a right-sided hemicolectomy due to a histopathological proven or suspected colon carcinoma from April 2009 to December 2016 at the University Hospital Wuerzburg. Patients were identified through the Wuerzburg Institutional Database (WID), a central data repository, which is expanded prospectively on a daily basis with clinical, operational, and research data. Data available within the WID includes patient demographics, histological diagnoses based on the “International Classification of Diseases” coding standards, physician and hospital billing data, inpatient admission and outpatient registration data, operating room procedures, laboratory results, and pharmacy records. The WID undergoes continuous cross-platform integration with the Comprehensive Cancer Registry. Additionally, inpatient and outpatient records of all identified patients were reviewed individually to confirm the histological diagnosis of colorectal adenocarcinoma, the type and duration of the administered chemotherapy, location of metastatic disease at presentation, and the vital status at last follow-up. All patients were treated according to national/international guidelines and discussed in a multidisciplinary team meeting.

### Statistics

Continuous variables were expressed as median with range or mean ± standard deviation (SD) and categorical variables in percent. Student *t* test or chi-squared test was performed to compare lap- vs. open-related differences. All results were considered significant with *p* < 0.05.

### Ethics

The ethics committee of the University of Wuerzburg has approved the studies from the WID due to its retrospective and anonymized nature (#20170918 01). The head of the board for internal data requests granted permission to access data from the registry.

## Results

Oncological right-sided hemicolectomy with central vessel ligation and systematic lymphadenectomy (Hohenberger procedure) was systematically introduced in our hospital from 1 October 2008 onwards. After a 6-month validation phase, we started to include patients in this study.

From 1 April 2009 to 31 December 2016, a total of 279 patients with suspected right-sided colon cancer (histologically proven and endoscopically not resectable adenoma with suspected cancer; cecum and C. ascendens) underwent oncological right-sided colon resection. The median age was 73.4 years (range 17.4–92.7) and 46.6% (*n* = 130) were female. The final histopathology showed that 44 (15.8%) patients had an adenoma, 47 (16.8%) were in UICC stage I, 77 (27.6%) in UICC stage II, 70 (25.1%) in UICC stage III, and 41 (14.7%) in UICC stage IV. In 272 cases, the number of retrieved lymph nodes was reported in the pathological report. In seven cases (patients with adenoma), the final pathological report did not mention the number of retrieved lymph nodes. The median of resected lymph nodes was 29 (average 31.8 ± 13.2; range 10–73). Table 1 summarizes the patient characteristics and tumor specific data.

**Table 1:** Patients characteristics of all right-sided colon resections

Characteristic	Patients total (*n* = 279)	
	No.	%
Sex		
Male	149	53.4
Female	130	46.6
Age [years]		
Median	73.4	
Average ± SD	70.6	
Range	17.4–92.7	
BMI		
Median	25.7	
Average ± SD	26.2	
Range	16.4–49.3	
ASA		
I	6	2.2
II	139	49.8
III	121	43.4
IV	13	4.6
pUICC stage		
0	44	15.8
I	47	16.8
II	77	27.6
III	70	25.1
IV	41	14.7
pT stage		
0	44	15.8
1	24	8.6
2	32	11.4
3	127	45.6
4	52	18.6
pN stage		
0	182	65.2
1	48	17.2
2	49	17.6
pM stage		
0	238	85.3
1	41	14.7
Number lymph nodes resected (*n* = 272)		
Median	29	
Average ± SD	31.8	
Range	10–73	
OP time [min]		
Median	142	
Average ± SD	152.2	
Range	61–443	
LOS [days] (*n* = 254)		
Median	12	
Average ± SD	15.5	
Range	2–83	
ICU [days] (*n* = 252)		
Median	1	
Average ± SD	2.6	
Range	0–41	

**Fig. 1:**
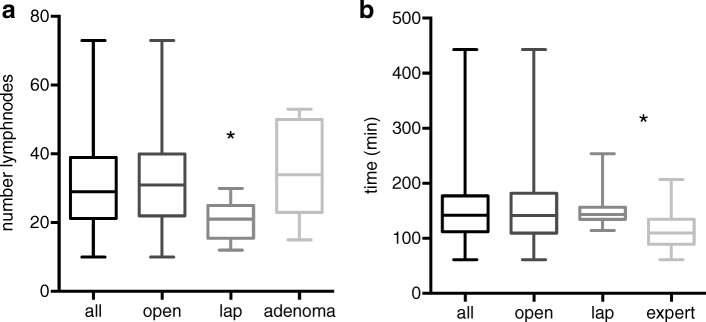
**a** Box plot analysis of lymph nodes depending on the operation method or histology (*p < 0.01 lap vs. each other procedure/subgroup). **b** Box plot analysis of the operation time depending on the type of operation or the surgeon’s experience (*p < 0.01 lap vs. experienced surgeon)

Twenty-four of the 279 patients (8.6%) had a laparoscopic resection (Table 2). These patients were significantly younger and had a significantly lower ASA score, significantly smaller tumors (T-categories), and lower UICC stage than patients who were operated by an open procedure. Postoperative ICU stay and total length of stay were significantly shorter in laparoscopically operated patients, whereas the operation time itself did not differ between both groups. The overall mortality rate was 1.4% (four patients). All of them did undergo an open procedure. The reoperation rate was 20.0% in the open expert group vs. 4.2% (*p* < 0.05). To our surprise and in contrary to the current published literature, the number of retrieved lymph nodes was significantly lower in laparoscopically operated patients compared to patients undergoing an open operation (median (range) 31 (10–73) vs. 21 (12–30); average 32.7 ± 13.3 vs. 21 ± 5.3; *p* < 0.001) (Fig. 1a).

**Table 2:** Patient characteristics in relation to the type of surgical procedure (open vs. laparoscopic)

Characteristic	Patients total (*n* = 255)		Patients total lap (*n* = 24)		*p* value
	No.	%	No.	%	
Sex					
Male	118	46.3	12	50.0	n.s.
Female	137	53.7	12	50.0	
Age [years]					
Median	74.4		61.3		< 0.01
Average ± SD	71.2 ± 12.6		63.9 ± 13.7		
Range	18.7–92.7		17.4–85.7		
BMI					
Median	25.8		24.9		n.s.
Average ± SD	26.3 ± 4.6		25.6 ± 5.5		
Range	16.4–49.3		19.3–47		
ASA					
I	6	2.4	0	0.0	< 0.01
II	119	47.7	20	83.3	
III	117	45.9	4	16.7	
IV	13	5.1	0	0.0	
pUICC stage					
0	23	9.0	21	87.5	< 0.001
I	44	17.3	3	12.5	
II	77	30.3	0	0.0	
III	70	27.5	0	0.0	
IV	41	16.1	0	0.0	
pT stage					
0	23	9.0	21	87.5	< 0.001
1	21	8.2	3	12.5	
2	33	12.9	0	0.0	
3	127	49.8	0	0.0	
4	51	20.0	0	0.0	
pN stage					
0	157	61.6	24	100.0	< 0.001
1	49	19.2	0	0.0	
2	49	19.2	0	0.0	
pM stage					
0	214	83.9	24	100.0	0.03
1	41	16.1	0	0.0	
Number lymph nodes resected					
Median	31		21		< 0.001
Average ± SD	32.7 ± 13.3		21 ± 5.3		
Range	10–73		12–30		
OP time [min]					
Median	141		143		n.s.
Average ± SD	152.4 ± 62.0		149.4 ± 30.2		
Range	61–443		114–254		
LOS [days]					
Median	12		7.5		0.005
Average ± SD	16.1 ± 11.6		9.3 ± 4.9		
Range	2–83		5–28		
ICU [days] (*n* = 252)					
Median	1		0		0.03.
Average ± SD	2.8 ± 5.3		0.375 ± 0.65		
Range	0–41		0–2

This comparison has some bias as the open group included all patients, also those with prior operations, additional simultaneous procedures as liver resection or HIPEC, more advanced tumor stages, and were performed by surgeons with different levels of experience. Due to these differences, several subgroup analyses have been performed (Table 3).

**Table 3:** Subgroup analysis of patients, who would potentially qualify for laparoscopic surgery vs. patients, who underwent laparoscopic surgery

Characteristic	Patients total open (*n* = 114)		Patients total lap (*n* = 24)		*p* value
	No.	%	No.	%	
Sex					
Male	66	57.9	12	50.0	n.s.
Female	48	42.1	12	50.0	
Age [years]					
Median	71.4		61.3		0.01
Average ± SD	71.4 ± 12.6		63.9 ± 13.7		
Range	18.7–90.3		17.4–85.7		
BMI					
Median	24.8		24.9		n.s.
Average ± SD	25.7 ± 4.4		25.6 ± 5.5		
Range	17.1–37.6		19.3–47		
ASA					
I	2	1.8	0	0.0	n.s.
II	66	57.9	20	83.3	
III	42	36.8	4	16.7	
IV	4	3.5	0	0.0	
pUICC stage					
0	12	10.5	21	87.5	< 0.001
I	21	18.4	3	12.5	
II	42	36.8	0	0.0	
III	35	30.7	0	0.0	
IV	4	3.5	0	0.0	
pT stage					
0	12	10.5	21	87.5	< 0.001
1	9	7.9	3	12.5	
2	16	14.0	0	0.0	
3	59	51.8	0	0.0	
4	18	15.8	0	0.0	
pN stage					
0	78	68.4	24	100.0	0.006
1	19	16.7	0	0.0	
2	17	14.9	0	0.0	
pM stage					
0	110	96.5	24	100.0	n.s.
1	4	3.5	0	0.0	
Number lymph nodes resected					
Median	34.5		21		< 0.001
Average ± SD	35.9 ± 13.1		21 ± 5.3		
Range	13–73		12–30		
OP time [min]					
Median	109.5		143		< 0.001
Average ± SD	114.1 ± 31.5		149.4 ± 30.2		
Range	61–207		114–254		
LOS [days]					
Median	11		7.5		0.013
Average ± SD	14.87 ± 10.6		9.3 ± 4.9		
Range	4–56		5–28		
ICU [days] (*n* = 252)					
Median	1		0		n.s.
Average ± SD	1.8 ± 4.5		0.375 ± 0.65		
Range	0–41		0–2		

First, we defined a group of patients with open surgery, who could have potentially also been operated laparoscopically. This group only included patients without additional procedures and excluded patients with T4 tumors or prior operations. Out of this group, we selected patients who were operated by experienced laparoscopic and open colorectal surgeons, as the laparoscopic hemicolectomy is also only performed by experienced laparoscopic surgeons. When comparing this subgroup of 114 patients to the group of laparoscopically operated patients, open-operated patients were still significantly older, in an advanced tumor stage, and the length of stay was significantly longer. Operation time in the open group was significantly shorter (114.1 ± 31.5 vs. 149.4 ± 30.2; *p* < 0.001), and significantly, more lymph nodes were retrieved (34.5 vs. 21; *p* < 0.001). Both groups did not differ regarding postoperative death whereas reoperation rate was still significantly higher in the open group (15.8 vs. 4.2%; *p* < 0.05).

Second, most patients who underwent laparoscopic right-sided colon resection had an adenoma in the final histopathological results (UICC0 = 87.5%; UICCI = 12.5%). It can be speculated that in definitive adenoma, the pathologist reports less lymph nodes as lymph node metastasis is not to be expected. To rule out this bias, we performed a subgroup analysis comparing solely patients with suspected cancer but which turned out as adenoma in the final histopathological report who underwent open surgery (*n* = 23) to those who underwent laparoscopic surgery (*n* = 21). In this analysis, patients undergoing open procedures were again significantly older (71.7 ± 8.4 vs. 60.7 ± 14.2; *p* = 0.01) and had a significantly longer hospital stay (17.8 ± 14.7 vs. 9.3 ± 5.2; *p* = 0.01). But still, in the open procedure, significantly more lymph nodes were reported by the pathologist (34.1 ± 13.4 vs. 22 ± 5.4; *p* < 0.001) (Table 4).

**Table 4:** Comparison of patients with adenoma in definitive histopathology vs. laparoscopic adenoma surgery group

Characteristic	Patients open-operated for adenoma (*n* = 23)		Patients lap operated for adenoma (*n* = 21)		*p* value
	No.	%	No.	%	
Sex					
Male	17	73.9	9	42.9	0.05
Female	6	26.1	12	57.1	
Age [years]					
Median	71.7		60.7		0.01
Average ± SD	70.2 ± 8.4		63.5 ± 14.2		
Range	49.5–83.7		17.4–85.7		
BMI					
Median	25,7		24.9		n.s.
Average ± SD	25.8 ± 4.2		25.8 ± 5.9		
Range	18.4–32.9		19.3–47		
ASA					
I	1	4.4	0	0.0	n.s.
II	15	65.2	18	85.7	
III	6	26.1	3	14.3	
IV	1	4.4	0	0.0	
pUICC stage					
0	23	100	21	100	n.s.
I	0	0	0	0.0	
II	0	0	0	0.0	
III	0	0	0	0.0	
IV	0	0	0	0.0	
pT stage					
0	23	100	21	100	n.s.
1	0	0	0	0.0	
2	0	0	0	0.0	
3	0	0	0	0.0	
4	0	0	0	0.0	
pN stage					
0	23	100.0	21	100.0	n.s.
1	0	0	0	0.0	
2	0	0	0	0.0	
pM stage					
0	23	100.0	21	100.0	n.s.
1	0	0	0	0.0	
Number lymph nodes resected (*n* = 21)					
Median	34		21.6		< 0.001
Average ± SD	34.1 ± 13.4		22 ± 5.4		
Range	15–53		12–30		
OP time [min]					
Median	124		147		n.s.
Average ± SD	137.2 ± 43.2		142 ± 30.2		
Range	70–21		114–254		
LOS [days] (*n* = 24)					
Median	12		7.5		0.01
Average ± SD	17.8 ± 14.7		9.3 ± 5.2		
Range	2–56		5–28		
ICU [days] (*n* = 24)					
Median	0		0		0.046
Average ± SD	1.7 ± 3.1		0.43 ± 0.68		
Range	0–14		0–2		

## Discussion

The most common carcinoma of the gastrointestinal tract is the colorectal carcinoma. In the last decades, after the introduction of the surgical resection according to the embryonic fascias, the patient survival has improved considerably. During the same period of time, laparoscopy has been established in abdominal surgery. Multiple randomized studies and meta-analysis have shown that laparoscopic surgery for left-sided colon and rectum carcinoma has advantages in the short-time course and is not inferior to open surgery in the oncological long term [9, 14–18].

To what extent the CME in right-sided colon carcinoma can be performed laparoscopically with equal results compared to the open procedure has not yet been examined. Up to date, there are no new randomized, multicenter trials comparing the laparoscopic to the open CME in right-sided colon carcinoma.

In our non-randomized trial, laparoscopically operated patients were significantly younger and had a significantly lower ASA score and a significantly lower UICC stage. Therefore, the length of hospital stay and the length of time spent in an intensive care unit were significantly shorter, consistent with the current literature. However, significantly more lymph nodes were resected in the open surgery, independent of the level of experience of the surgeon and independent of the final histological stage.

After the introduction of the CME for right-sided colon carcinoma in 2009, the number of resected lymph nodes increased. This lead to an increased detection of lymph node metastasis in about 20% of patients with otherwise normal lymph nodes, thus leading to a “stage migration.” One can postulate that these patients might have suffered a recurrence if they had not been operated with CME [19]. A population-based study in Denmark showed that the disease-free survival is significantly increased when patients are operated with CME and that significantly more lymph nodes are resected (median 19 vs. 34) [6]. The number of resected lymph nodes can, thus, be used as a surrogate parameter for the oncological outcome. Our study showed 31 resected lymph nodes in the median in the open-operated patients. These results are consistent with the results from the working group of Prof. Hohenberger and those from the abovementioned Danish study [5, 6]. Interestingly, the number of resected lymph nodes in the laparoscopically operated patients is significantly lower (median of 21 lymph nodes). These results are comparable to a study of West et al., which showed an identical CME quality in laparoscopically and open-operated patients, though showing a significantly lower number of resected lymph nodes in the laparoscopic group comparable to patients in whom no central lymph node dissection had been performed [20]. A number of European studies, comparing the laparoscopic to the open resection of right-sided colon carcinoma, have similarly shown about 20 resected lymph nodes in the laparoscopic group [12, 13, 21, 22]. A few of these studies also reported 20 resected lymph nodes in the open operated patient group and, thus, reason that laparoscopy and open surgery are equal concerning the oncological outcome and the number of resected lymph nodes. When comparing these results to those of our study or of the studies from Denmark or Prof. Hohenberger, the equality of the oncological outcome must be critically questioned. In contrast, Asian studies were able to show a higher number of resected lymph nodes in the laparoscopically operated patients (25–30 lymph nodes). The different physiognomy might play a role leading to these results. A summary of the current literature is shown in Table 5.

**Table 5:** Summary of current literature comparing the laparoscopic to the open resection of right-sided colon carcinoma after the introduction of CME

Author	Year	Region	Procedure	Cases	Retrieved LN	Range
Bae [23]	2014	Asia	Open RH	85	28	8–79
			Lap RH	85	27	8–62
Gouvas [24]	2012	Europe	Open RH	9	30	17–60
			Lap RH	7	33	21–46
Kang [25]	2016	Asia	Open RH	33	31.8 ± 16.9	
			Lap RH	43	32.3 ± 16.5	
Kim [26]	2016	Asia	Open RH	99	31 ± 12	
			Lap RH	116	27 ± 11	
Li [27]	2012	Asia	Open RH	74	20.7 ± 11.4	
			Lap RH	71	18.7 ± 12	
Luca [22]	2011	Europe	Open RH	102	25.4	8–74
			Robotic RH	33	26.6	15–46
Sheng [28]	2012	Asia	Open RH	57	14 ± 5.6	
			Lap RH	59	14.4 ± 5.4	
Sim [29]	2013	Asia	Lap RH	16	31.5 ± 18.0	
			Open RH	33	36.1 ± 24.1	
Tiefenthal [13]	2015	Europe	Open RH	123	17.7 ± 6.2	
			Lap RH	169	18.9 ± 8.5	
Zimmermann [12]	2016	Europe	Open RH	94	16.0	7–35
			Lap RH	94	17.0	8–38

Looking at the pathological tumor stage, the patients operated laparoscopically had significantly smaller tumors and a significantly lower UICC stage. Here, a bias from the pathologists’ side can be postulated, stating that less lymph nodes are examined if the tumor is not malignant. This is not the case in our pathology department. All sections are equally examined independent of the type of tumor. Open-operated patients with an adenoma also show significantly more resected lymph nodes compared to those operated laparoscopically.

The duration of the open and laparoscopic surgery does not differ significantly. However, the open right-sided hemicolectomy is a procedure also performed by surgeons in training, whereas the laparoscopic right-sided hemicolectomy is only performed by few expert surgeons. Also, the open procedures also often include further resections, such as liver resections, extended resections, or HIPEC. A subgroup analysis showed that the duration of the operation of open right-sided hemicolectomy in patients without previous surgeries performed by an expert surgeon is significantly shorter.

Our study is a retrospective study, in which a full explanation of the selected surgical procedure in each patient is not completely traceable, thus limiting the study. Furthermore, the number of resected lymph nodes is used as a surrogate parameter for the oncological outcome, but validated data concerning the patient survival was not examined. In addition, the number of laparoscopically operated patients was low, and patients showed a significantly lower UICC stage, thus limiting the validity of a potential survival advantage.

## Conclusion

Up to date, the data concerning the laparoscopic right-sided hemicolectomy remains unclear. Due to the clear survival benefit after the introduction of the CME with central ligation of the vessels, the CME is strongly recommended. The laparoscopic right-sided hemicolectomy should only be performed in controlled studies until the oncological non-inferiority can be proven. For now, the short-term disadvantages of the open surgery must be accepted. This is especially relevant for patients with an assumed adenoma or a low UICC stage because this patient group benefits the most from the CME.
